# Alteration of Mitochondrial Ca^2+^ Fluxes by Kaempferol and CGP-37157 Regulates Ca^2+^ Oscillations in Human Alveolar Type 2 A549 Cells

**DOI:** 10.33549/physiolres.935636

**Published:** 2026-02-01

**Authors:** King-Chuen WU, Cing-Yu CHEN, Lian-Ru SHIAO, Zih-He YANG, Chin-Min CHUANG, Yuk-Man LEUNG

**Affiliations:** 1 Department of Anesthesiology, Chang Gung Memorial Hospital, Chiayi, Taiwan; 2 Department of Cosmetic Science, Providence University, Taichung, Taiwan; 3 School of Pharmacy, China Medical University, Taichung, Taiwan; 4 Department of Physiology, China Medical University, Taichung, Taiwan; 5 Department of Emergency Medicine, China Medical University Hospital, Taichung, Taiwan

**Keywords:** Mitochondria, Ca^2+^ oscillations, Mitochondrial Ca^2+^ uniporter, Mitochondrial Na^+^, Ca^2+^ exchanger, A549 cells

## Abstract

Mitochondria participate in regulating cytosolic Ca^2+^ signaling by their Ca^2+^ handling *via* mitochondrial Ca^2+^ uniporter (MCU) and mitochondrial Na^+^/Ca^2+^ exchanger (mitoNCX). In this study, we examined how agonist-triggered cytosolic Ca^2+^ oscillations in human alveolar type 2 A549 cells were affected by an MCU inhibitor (MCU-i4), MCU activator (kaempferol) and mitoNCX inhibitor (CGP-37157). Whilst inhibition of MCU did not significantly repress Ca^2+^ oscillations, MCU activation by kaempferol considerably dampened oscillatory activities. Inhibition of mitochondrial Ca^2+^ efflux by CGP-37157 also suppressed Ca^2+^ oscillations; the suppressive effects of kaempferol and CGP﷓37157 were not additive. Both kaempferol and CGP-37157 caused a rise in mitochondrial matrix Ca^2+^ level, but their effects were not additive. Taken together, our results suggest Ca^2+^ oscillations in alveolar type 2 A549 cells were regulated by stimulating Ca^2+^ uptake into, and preventing Ca^2+^ efflux from, the mitochondria, with both cases resulting in disturbed Ca^2+^ traffic and Ca^2+^ accumulation in the mitochondrial matrix.

One of the functions of mitochondria is its regulation of cytosolic Ca^2+^ signaling by its uptake of Ca^2+^ *via* mitochondrial Ca^2+^ uniporter (MCU) [1]. MCU is a complex comprising the MCU channel and accessory proteins such as mitochondrial Ca^2+^ uniporter regulator 1 and mitochondrial Ca^2+^ uptake proteins (MICU-1, -2 and -3) [1]. In gall bladder smooth muscle, block of mitochondrial Ca^2+^ uptake reduces frequency of Ca^2+^ waves [2]. By contrast, in cardiac myocytes, inhibition of mitochondrial Ca^2+^ uptake by Ru360 increases amplitude and frequency of Ca^2+^ waves [3]. Effects of MCU stimulation on Ca^2+^ signaling are also variable. In rat ventricular myocytes, increased mitochondrial Ca^2+^ uptake by kaempferol promotes Ca^2+^ wave generation under β-adrenergic stimulation [4]. Activation of mitochondrial Ca^2+^ uptake by kaempferol initially enhances but later suppresses histamine-elicited Ca^2+^ oscillations in HeLa cells and human fibroblasts [5].

The modulations of Ca^2+^ waves and oscillations by MCU inhibition and activation are therefore variable and hitherto have not been fully understood. Nonetheless, MCU-modulating drugs are of potential therapeutic values. In a traumatic brain injury rat model, treatment with Ru360, an MCU inhibitor, significantly alleviates oxidative stress and improves sensorimotor behavioral recovery [6]. MCU activators also offer neuroprotective effects [7].

Lung expansion causes synchronous cytosolic Ca^2+^ oscillations in all alveolar cells and surfactant secretion in type 2 cells; the exocytosis rate is positively correlated to Ca^2+^ oscillation frequency [8]. In this study, we examined how cytosolic Ca^2+^ oscillations in human alveolar type 2 A549 cells were affected by drugs which modulate mitochondrial Ca^2+^ fluxes: MCU inhibition and activation, respectively, by MCU-i4 and kaempferol; and effects of inhibition of mitochondrial Na^+^/Ca^2+^ exchanger (mitoNCX) by CGP-37157.

Dulbecco's modified Eagle's medium (DMEM), fetal calf serum, and tissue culture reagents were purchased from Invitrogen Corporation (Carlsbad, CA, USA). ATP and cyclopiazonic acid (CPA) were from Sigma-Aldrich chemical Co. (St. Louis, MO, USA). MCU-i4, kaempferol and CGP-37157 were from Tocris Bioscience (Bristol, U.K.). Except for ATP (dissolved in distilled water; 100 mM as stock), all other chemicals mentioned above were dissolved in DMSO as stock solutions (30 or 50 mM). All other chemicals of reagent grade were obtained from Sigma-Aldrich. A549 cells were cultured in RPMI-1640 medium supplemented with 10 % FBS, 2 mM L-glutamine, 100 U/ml penicillin, and 100 μg/ml streptomycin at 37 °C with humidified 5 % CO_2_.

Measurement of cytosolic Ca^2+^ concentration was conducted as previously described [9,10]. Cells were incubated with 5 μM fura-2 AM (Invitrogen) for 1 h at 37 °C and subsequently washed in bath solution (mM): 140 NaCl, 4 KCl, 1 MgCl_2_, 2 CaCl_2_, 10 HEPES (NaOH was used to adjust pH to 7.4). Cells were excited with 340 nm and 380 nm alternately (frequency of switching = 1 Hz) with the aid of an optical filter changer (Lambda 10-2, Sutter Instruments, Novato, CA). Emission was collected at 500 nm and data were captured with a CCD camera (CoolSnap HQ2, Photometrics, Tucson, AZ, USA) connected to a Nikon TE2000-U microscope. Microscope magnification was 400×. ATP (10 μM) was used to trigger cytosolic Ca^2+^ oscillations. Results were analyzed by an MAG Biosystems Software (Sante Fe, MN). Experiments were carried out at 25 °C. 340/380 ratio changes were measured and analyzed at a region of interest of single cells in one experiment; the same experimental protocols were repeated a few more times to obtain the mean.

Cells were incubated with 5 μM Rhod-2 AM (Invitrogen, Carlsbad, CA) for 1 h at 25 °C and then washed. Cells were permeabilized and washed with a digitonin (30 μM)-containing intracellular solution which contained (mM): 140 KCl, 8 NaCl, 1 MgCl_2_, 1.85 EGTA, 1 CaCl_2_, 10 HEPES, and 8 MgATP (pH 7.25 adjusted with KOH; free [Ca^2+^] was calculated to be 114 nM using the “Ca-EGTA Calculator TS version 1.3”, a free online program provided by University of California, Davis). Cells were trypsinized, dispersed and washed in intracellular solutions. The cells were then treated with different agents for 5 min before subject to fluorescence-activated cell sorting and then analyzed using a FACS Canto flow cytometer system (BD Biosciences, San Jose, CA, USA). Excitation and emission wavelengths were set at 488 and 576 nm, respectively. Data were analyzed by BD FACSDIVA™ software (BD Biosciences).

Results were presented as means ± S.E.M. When 2 groups were compared, paired or unpaired Student’s *t*-test was used when appropriate. Multiple groups were analyzed by one-way analysis of variance (ANOVA), followed by Tukey’s HSD *post hoc* test. Statistical significance is reached when P is <0.05.

Continuous Ca^2+^ transfer from endoplasmic reticulum (ER) *via* IP_3_ receptor (IP_3_R) and ryanodine receptor (RYR) to mitochondria (*via* MCU) is necessary for mitochondrial functions [11]. As reported in our previous study [9], addition of ATP to A549 cells triggered Ca^2+^ oscillations in the majority of cells. Inhibition of MCU by MCU-i4 did not significantly affect the percentage of oscillating cells (Fig. 1A, B), but moderately affected Ca^2+^ oscillation frequency: the latter was reduced by 21.4 % by 10 μM MCU-i4 (P=0.028; Fig. 1C).

By contrast, pretreatment with kaempferol (MCU activator), strongly suppressed Ca^2+^ oscillations (Fig. 1D). Kaempferol reduced % oscillating cells and oscillation frequencies (P<0.001; Fig. 1E, F). Intriguingly, with kaempferol treatment, the first peaks of ATP-triggered Ca^2+^ signal were not smaller but indeed slightly higher than those of the control group (P<0.01; Fig. 1G).

Emptiness of ER Ca^2+^ stores triggers Ca^2+^ entry from the extracellular space into the cell, a term coined “store-operated Ca^2+^ entry” (SOCE) [12]. We showed earlier that SOCE was necessary to support Ca^2+^ oscillations in A549 cells [9]. Therefore, we examined if kaempferol affected SOCE. After Ca^2+^ pool discharge by cyclopiazonic acid, Ca^2+^ influx upon Ca^2+^ replenishment was not affected by kaempferol, suggesting kaempferol did not suppress Ca^2+^ oscillations by blocking SOCE (data not shown).

If kaempferol inhibited Ca^2+^ oscillations by increasing mitochondrial Ca^2+^ level, then prevention of Ca^2+^ efflux *via* inhibiting mitoNCX would be expected to suppress Ca^2+^ oscillations. The effects of kaempferol, CGP-37157 (selective mitoNCX inhibitor), or a combination of both agents, were examined (Fig. 2A). Kaempferol suppressed both % oscillating cells and oscillation frequency whilst CGP-37157 only reduced oscillation frequency (P<0.01; Fig. 2B, C). Unexpectedly, suppressive effects of kaempferol and CGP-37157 were not additive. There were also no additive effects of kaempferol and CGP-37157 in enhancing the first peak of ATP-triggered Ca^2+^ signal (Fig. 2D). We also examined whether kaempferol and CGP-37157 affected mitochondrial Ca^2+^ levels (Fig. 2E). These drugs, either alone or in combination, caused a significant rise in mitochondrial Ca^2+^ level, but their effects were not additive.

The mechanisms of Ca^2+^ oscillations have been extensively studied (for a review see [12]). Agonist-triggered generation of IP_3_ activates IP_3_R on ER to release Ca^2+^; the resultant elevated concentration of cytosolic Ca^2+^ acts as a co-agonist of IP_3_R and promotes IP_3_-induced Ca^2+^ release (IICR). Meanwhile, the empty state of ER induces SOCE, causing further elevation in cytosolic Ca^2+^. The latter subsequently inactivates IICR, stimulates ER Ca^2+^ pump (to re-sequester Ca^2+^) and plasma membrane Ca^2+^ pump to extrude Ca^2+^. The decline of cytosolic Ca^2+^ back to resting level removes inhibition of IP_3_R and allows the latter to be reactivated by IP_3_ to begin the cycle anew.

CGP-37157 was effective in reducing oscillation frequency (Fig. 2), suggesting Ca^2+^ efflux from mitochondria is important in maintaining Ca^2+^ oscillations. This is in agreement with a previous report that mitochondrial Ca^2+^ release *via* mitoNCX sustains Ca^2+^ oscillations [13]. The effect of MCU-i4 on Ca^2+^ oscillations, however, was only minimal (Fig. 1), suggesting reduced mitochondrial Ca^2+^ uptake was much less pivotal than reduced mitochondrial Ca^2+^ efflux in regulating oscillations.

Kaempferol was much more efficacious than MCU-i4 and CGP-37157 in suppressing Ca^2+^ oscillations. Our previous study suggests blockade of SOCE sufficed to suppress Ca^2+^ oscillations in A549 cells [9]. However, the inability of kaempferol to block SOCE (not shown) suggests it did not suppress Ca^2+^ oscillations by blocking SOCE. Furthermore, kaempferol suppression of Ca^2+^ oscillations was unlikely attributed to inhibition of purinergic signaling, as the first peak of ATP-triggered Ca^2+^ signal was even slightly higher in the presence of kaempferol (and CGP-37157 as well) (Fig. 1 and Fig. 2). The reason was unclear. Kaempferol- and CGP-37157-treated cells had mitochondrial Ca^2+^ overload (Fig. 2E). The higher first peaks of Ca^2+^ oscillations of kaempferol- and CGP-37157-treated cells may be due to Ca^2+^ efflux from over-loaded mitochondria *via* the Ca^2+^/H^+^ exchanger [14].

A tantalizing explanation to kaempferol suppression is that it increased Ca^2+^ loading in the mitochondrial matrix; if it is true, CGP-37157 should be able to amplify this effect by preventing mitochondrial Ca^2+^ efflux. However, CGP-37157 and kaempferol did not add up in their enhancing effect on mitochondrial matrix Ca^2+^ accumulation (Fig. 2E); the reason was uncertain but could be due to near-saturation of matrix Ca^2+^ level. The observation that a combination of CGP-37157 and kaempferol did not have an additive effect in suppressing Ca^2+^ oscillations was in concordance with the non-additive effects of these two agents on mitochondrial matrix Ca^2+^ accumulation.

As Ca^2+^ is a co-agonist of IP_3_R and Ca^2+^ dependence of IICR has a bell-shaped relationship [15], one possible hypothesis is that kaempferol, by activating MCU, accelerated the Ca^2+^ fluxes in mitochondria-associated membranes (MAM; region of ER tethering to mitochondria), reducing the local Ca^2+^ accumulation which would otherwise provide a positive feedback on IICR [16]. MCU knockdown, however, reduces Ca^2+^ buffering, resulting in Ca^2+^-dependent inactivation of IP_3_R and eventually run down of oscillations [17]. These contradictory results of MCU manipulation on Ca^2+^ oscillations (including those mentioned in the Introduction) is difficult to explain. One possible explanation is the variable thickness of the MAM (which determines the distance between IP_3_R and MCU): such thickness may vary in different cell types and is modulated by metabolic or disease states [18]. Given the variable distance between IP_3_R and MCU, and the bell-shaped Ca^2+^ dependence of IICR, changes of micro-domain Ca^2+^ concentration (at IP_3_R) due to MCU manipulation (activation or block) could be either stimulatory or inhibitory. Accurate measurement of Ca^2+^ changes at such MAM micro-domains is needed to gain further insight into how Ca^2+^ oscillations are modulated by MCU manipulation.

The implication of kaempferol-induced inhibition of Ca^2+^ oscillations is two-fold. First, pharmacological activation of MCU offers beneficial effects [7]. Further development of MCU activators as therapeutic agents should be cautious in view of their suppression of Ca^2+^ oscillations. Second, kaempferol-induced inhibition of Ca^2+^ oscillations suggests any increased intrinsic MCU activity may affect Ca^2+^ oscillations; these situations may arise due to MCU overexpression [19] or MICU-1 loss-of-function [20].

In conclusion, ATP-triggered Ca^2+^ oscillations in A549 cells were regulated by stimulating Ca^2+^ uptake into, and preventing Ca^2+^ efflux from, the mitochondria, with both cases resulting in disturbed Ca^2+^ traffic and Ca^2+^ accumulation in the mitochondrial matrix.

## Figures and Tables

**Fig. 1:**
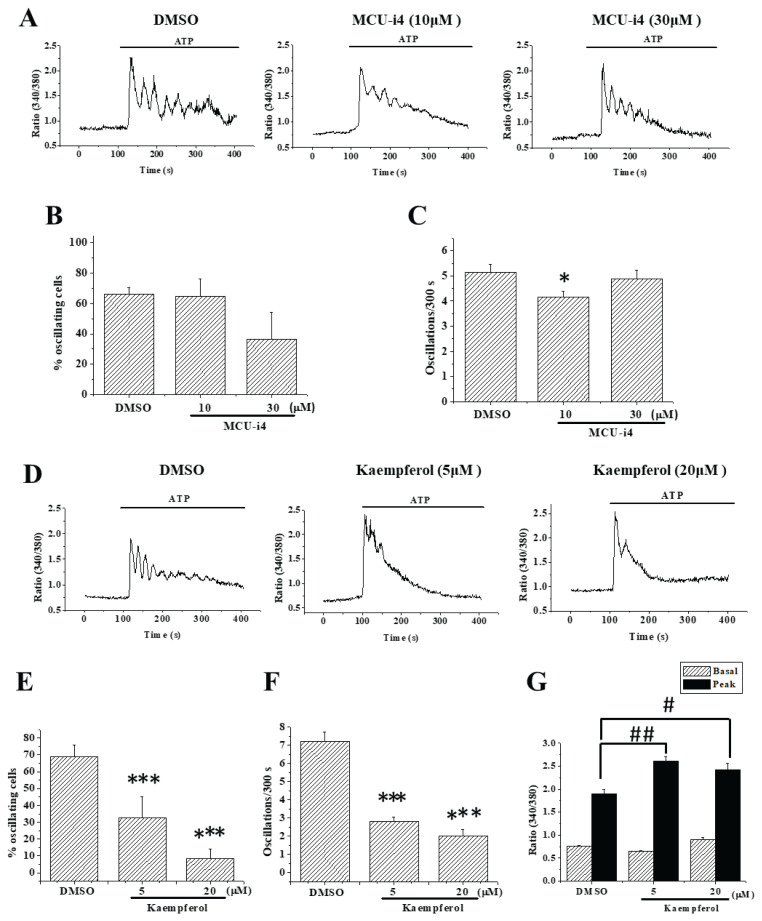
Effects of MCU-i4 and kaempferol on ATP-triggered Ca^2+^ oscillations. (**A**) A549 cells in Ca^2+^-containing bath solution were pre-treated with DMSO, 10 or 30 μM MCU-i4 for 8 min and then stimulated by 10 μM ATP. (**B**–**C**) * significantly different from the control (P=0.028). (**D**) A549 cells in Ca^2+^-containing bath solution were pre-treated with DMSO or kaempferol for 8 min and then stimulated by 10 μM ATP. (**E–F**) *** different from control (P<0.001). (**G**) shows averaged basal/peak values of first ATP-triggered Ca^2+^ signal. **^#^** P<0.05 and **^##^** P<0.01 indicate significant difference from control. Results are mean ± S.E.M.; each group had 49–98 cells from 5–11 separate experiments.

**Fig. 2:**
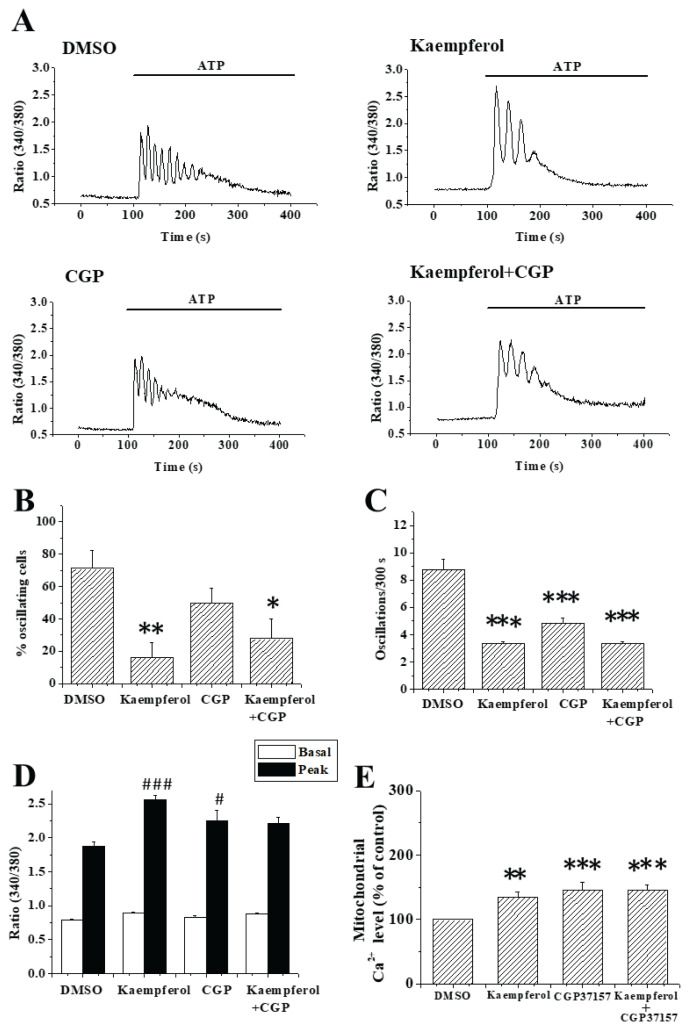
Effects of kaempferol and CGP-37157 on Ca^2+^ oscillations and mitochondrial Ca^2+^ concentration. (**A**) A549 cells in Ca^2+^-containing bath solution were pre-treated with DMSO, 20 μM kaempferol, 10 μM CGP-37157 (CGP) or 20 μM kaempferol plus 10 μM CGP-37157 for 8 min before stimulated by 10 μM ATP. (**B–C**) * P<0.05, ** P<0.01 and *** P<0.001 indicate significant differences compared to the control. (**D**) shows averaged basal/peak values of first ATP-triggered Ca^2+^ signals. **^#^** P<0.05 and **^###^** P<0.001 indicate significant differences from control. Results are mean ± S.E.M.; each group had 38–66 cells from 3–6 separate experiments. (**E**) A549 cells were treated with DMSO, 20 μM kaempferol (kaemp), 10 μM CGP-37157 (CGP), or a combination of these two agents for 5 min and then subject to mitochondrial Ca^2+^ level measurement. ** P<0.01 and *** P<0.001 are significantly different from the control. Results are mean ± SEM from 3 independent experiments.

## References

[1] Alevriadou BR, Patel A, Noble M, Ghosh S, Gohil VM, Stathopulos PB, Madesh M (2021). Molecular nature and physiological role of the mitochondrial calcium uniporter channel. Am J Physiol Cell Physiol.

[2] Balemba OB, Bartoo AC, Nelson MT, Mawe GM (2008). Role of mitochondria in spontaneous rhythmic activity and intracellular calcium waves in the guinea pig gallbladder smooth muscle. Am J Physiol Gastrointest Liver Physiol.

[3] Zhao Z, Gordan R, Wen H, Fefelova N, Zang WJ, Xie LH (2013). Modulation of intracellular calcium waves and triggered activities by mitochondrial Ca flux in mouse cardiomyocytes. PLoS One.

[4] Hamilton S, Terentyeva R, Kim TY, Bronk P, Clements RT, O-Uchi J, Csordás G, Choi BR, Terentyev D (2018). Pharmacological Modulation of Mitochondrial Ca2+ Content Regulates Sarcoplasmic Reticulum Ca2+ Release via Oxidation of the Ryanodine Receptor by Mitochondria-Derived Reactive Oxygen Species. Front Physiol.

[5] Vay L, Hernández-Sanmiguel E, Santo-Domingo J, Lobatón CD, Moreno A, Montero M, Alvarez J (2007). Modulation of Ca(2+) release and Ca(2+) oscillations in HeLa cells and fibroblasts by mitochondrial Ca(2+) uniporter stimulation. J Physiol.

[6] Chitturi J, Santhakumar V, Kannurpatti SS (2021). Traumatic brain injury metabolome and mitochondrial impact after early stage Ru360 treatment. Mitochondrion.

[7] Chitturi J, Santhakumar V, Kannurpatti SS (2019). Beneficial Effects of Kaempferol after Developmental Traumatic Brain Injury Is through Protection of Mitochondrial Function, Oxidative Metabolism, and Neural Viability. J Neurotrauma.

[8] Ashino Y, Ying X, Dobbs LG, Bhattacharya J (2000). [Ca(2+)](i) oscillations regulate type II cell exocytosis in the pulmonary alveolus. Am J Physiol Lung Cell Mol Physiol.

[9] Wu KC, Chen CY, Chuang CM, Shiao LR, Chan P, Leung YM (2022). Suppression of Ca2+ oscillations by SERCA inhibition in human alveolar type 2 A549 cells: rescue by ochratoxin A but not CDN1163. Life Sci.

[10] Chen CY, Chen YJ, Wang CA, Lin CH, Yeh JS, Chan P, Shiao LR, Leung YM (2025). Agonist-Triggered Ca2+ Release From Functionally Connected Endoplasmic Reticulum and Lysosomal Ca2+ Stores in bEND.3 Endothelial Cells. Physiol Res.

[11] Katona M, Bartók Á, Nichtova Z, Csordás G, Berezhnaya E, Weaver D, Ghosh A (2022). Capture at the ER-mitochondrial contacts licenses IP3 receptors to stimulate local Ca2+ transfer and oxidative metabolism. Nat Commun.

[12] Uhlén P, Fritz N (2010). Biochemistry of calcium oscillations. Biochem Biophys Res Commun.

[13] Ishii K, Hirose K, Iino M (2006). Ca2+ shuttling between endoplasmic reticulum and mitochondria underlying Ca2+ oscillations. EMBO Rep.

[14] Natarajan GK, Glait L, Mishra J, Stowe DF, Camara AKS, Kwok WM (2020). Total Matrix Ca2+ Modulates Ca2+ Efflux via the Ca2+/H+ Exchanger in Cardiac Mitochondria. Front Physiol.

[15] Missiaen L, Parys JB, Weidema AF, Sipma H, Vanlingen S, De Smet P, Callewaert G, De Smedt H (1999). The bell-shaped Ca2+ dependence of the inositol 1,4, 5-trisphosphate-induced Ca2+ release is modulated by Ca2+/calmodulin. J Biol Chem.

[16] Hajnóczky G, Hager R, Thomas AP (1999). Mitochondria suppress local feedback activation of inositol 1,4,5-trisphosphate receptors by Ca2+. J Biol Chem.

[17] Samanta K, Douglas S, Parekh AB (2014). Mitochondrial calcium uniporter MCU supports cytoplasmic Ca2+ oscillations, store-operated Ca2+ entry and Ca2+-dependent gene expression in response to receptor stimulation. PLoS One.

[18] Giacomello M, Pellegrini L (2016). The coming of age of the mitochondria-ER contact: a matter of thickness. Cell Death Differ.

[19] Hamilton S, Terentyeva R, Perger F, Hernández Orengo B, Martin B, Gorr MW, Belevych AE (2021). MCU overexpression evokes disparate dose-dependent effects on mito-ROS and spontaneous Ca2+ release in hypertrophic rat cardiomyocytes. Am J Physiol Heart Circ Physiol.

[20] Logan CV, Szabadkai G, Sharpe JA, Parry DA, Torelli S, Childs AM, Kriek M (2014). Loss-of-function mutations in MICU1 cause a brain and muscle disorder linked to primary alterations in mitochondrial calcium signaling. Nat Genet.

